# Complete Genome Sequence of the Sugar Cane Endophyte *Pseudomonas aurantiaca* PB-St2, a Disease-Suppressive Bacterium with Antifungal Activity toward the Plant Pathogen *Colletotrichum falcatum*

**DOI:** 10.1128/genomeA.01108-13

**Published:** 2014-01-23

**Authors:** Samina Mehnaz, Judith S. Bauer, Harald Gross

**Affiliations:** a Department of Biological Sciences, Forman Christian College University, Lahore, Pakistan; b Pharmaceutical Institute, Department of Pharmaceutical Biology, University of Tuebingen, Tuebingen, Germany

## Abstract

The endophytic bacterium *Pseudomonas aurantiaca* PB-St2 exhibits antifungal activity and represents a biocontrol agent to suppress red rot disease of sugar cane. Here, we report the completely sequenced 6.6-Mb genome of *P. aurantiaca* PB-St2. The sequence contains a repertoire of biosynthetic genes for secondary metabolites that putatively contribute to its antagonistic activity and its plant-microbe interactions.

## GENOME ANNOUNCEMENT

*Pseudomonas aurantiaca* PB-St2 (syn., *Pseudomonas chlororaphis* subsp. *aurantiaca* PB-St2) (1) is a member of the group of plant-beneficial *Pseudomonas* bacteria (2). It was originally isolated from surface-sterilized stems of sugar cane grown in Pakistan (3, 4) and exhibits antagonistic activity toward the fungal phytopathogen *Colletotrichum falcatum*, the causative agent of the red rot disease of sugar cane (*Saccharum* sp. hybrids) (5). Strain PB-St2 is known to produce several secondary metabolites, such as phenazines, homoserine lactones, hydrogen cyanide, siderophores of the hydroxamate type, the lipopeptide WLIP, and lahorenoic acids A to C (3, 4, 6).

The genome of *P. aurantiaca* PB-St2 was sequenced using a combination of next-generation sequencing platforms. Genomic DNA was first subjected to 50-cycle paired-end sequencing with an Illumina GAIIx (BaseClear, Leiden, The Netherlands). The *de novo* assembly of 4,870,658 reads (45-fold median coverage) was performed using the CLC Genomics Workbench 4.0, yielding 555 contigs and a total assembled length of 6,685,786 bp. To improve the quality of the sequence, the genome was additionally sequenced using the PacBio RS technology (10-kb library, 78,395 reads, 189 Mb, 2,412 kb average length). The data collected from the PacBio RS instrument were processed and filtered (SMRT analysis software suite, CLC Genomics Workbench version 6.0.1), and the results of both sequencing platforms were subsequently used to perform a *de novo* hybrid assembly. The contigs were linked and placed into superscaffolds based on the alignment of the PacBio continuous long reads (CLR). Alignment was performed with BLASR (7). From the alignment, the orientation, order, and distance were determined using a modified version of the SSPACE Premium scaffolder 2.3 (8). The final genome is scaffolded in 23 segments and includes 6,590,922 bases with a G+C content of 63.3%. These data are comparable to those of already-reported sequenced genomes of the closely related *P. chlororaphis* strains GP72 (9), 30-84, and O6 (10), which range from 6.6 to 6.9 Mb in size and have G+C contents of 62.8 to 63.1%.

The obtained sequence reflects a huge capacity to produce secondary metabolites on the genetic level. Gene cluster coding for the production of the cyclic lipopeptide WLIP (11), hydrogen cyanide (12), 2-hydroxyphenazine (13), and indole acetic acid (*iaaMH*) (14) was readily detected and annotated. Moreover, a candidate gene cluster coding for lahorenoic acids, biosynthetic pathways for a pyoverdine-like siderophore (15), the siderophore achromobactin (16, 17), the strong antifungal compound pyrrolnitrin (18), an incomplete mangotoxin cluster (*mgoABCD*, syn. *pvfABCD*, but *mboABCDEF* is absent) (19, 20), an orphan tetramodular nonribosomal peptide synthetase (NRPS) gene cluster, the osmolytes *N*-acetylglutaminylglutamine amide (NAGGN) and trehalose (21, 22), homoserine lactones (*phzIR, csaIR, hdtS*) (23, 24), two bacteriocin-like proteins (25), and genes encoding exoenzymes, such as chitinases (26) and AprA (27, 28), were found in the genome.

The genome sequence of PB-St2 not only revealed a true capacity to produce a multitude of secondary metabolites and exoenzymes but also provides a foundation for further understanding of the lifestyle, antagonistic properties, and respective modes of action of this strain and for biosafety assessments when introduced into agricultural environments.

### Nucleotide sequence accession numbers.

This whole-genome sequencing project has been deposited at DDBJ/EMBL/GenBank under the accession no. AYUD00000000. The version described in this paper is version AYUD01000000.
